# Syntactic Constraints and Individual Differences in Native and Non-Native Processing of Wh-Movement

**DOI:** 10.3389/fpsyg.2016.00549

**Published:** 2016-04-22

**Authors:** Adrienne Johnson, Robert Fiorentino, Alison Gabriele

**Affiliations:** ^1^Department of Education, Missouri Western State UniversitySt. Joseph, MO, USA; ^2^Neurolinguistics and Language Processing Laboratory, Department of Linguistics, University of KansasLawrence, KS, USA; ^3^Second Language Acquisition and Processing Laboratory, Department of Linguistics, University of KansasLawrence, KS, USA

**Keywords:** wh-dependencies, individual differences, self-paced reading, second language processing, counting span, reading span, islands, working memory

## Abstract

There is a debate as to whether second language (L2) learners show qualitatively similar processing profiles as native speakers or whether L2 learners are restricted in their ability to use syntactic information during online processing. In the realm of wh-dependency resolution, research has examined whether learners, similar to native speakers, attempt to resolve wh-dependencies in grammatically licensed contexts but avoid positing gaps in illicit contexts such as islands. Also at issue is whether the avoidance of gap filling in islands is due to adherence to syntactic constraints or whether islands simply present processing bottlenecks. One approach has been to examine the relationship between processing abilities and the establishment of wh-dependencies in islands. Grammatical accounts of islands do not predict such a relationship as the parser should simply not predict gaps in illicit contexts. In contrast, a pattern of results showing that individuals with more processing resources are better able to establish wh-dependencies in islands could conceivably be compatible with certain processing accounts. In a self-paced reading experiment which examines the processing of wh-dependencies, we address both questions, examining whether native English speakers and Korean learners of English show qualitatively similar patterns and whether there is a relationship between working memory, as measured by counting span and reading span, and processing in both island and non-island contexts. The results of the self-paced reading experiment suggest that learners can use syntactic information on the same timecourse as native speakers, showing qualitative similarity between the two groups. Results of regression analyses did not reveal a significant relationship between working memory and the establishment of wh-dependencies in islands but we did observe significant relationships between working memory and the processing of licit wh-dependencies. As the contexts in which these relationships emerged differed for learners and native speakers, our results call for further research examining individual differences in dependency resolution in both populations.

## Introduction

Research on the processing of wh-dependencies has found evidence that both native speakers and second language (L2) learners are able to utilize abstract syntactic information in the course of online processing (e.g., Aldwayan et al., 2010; Omaki and Schulz, 2011; Kim et al., 2015). The focus of these studies has been whether island constraints, which constrain the type of structures from which wh-extraction is possible (Ross, 1967; Chomsky, 1973, 1986), are respected in real time. For example, building on the seminal work of Stowe (1986), Aldwayan et al. (2010) examined whether L2 learners, similar to native speakers, would attempt to resolve wh-dependencies only in grammatically licensed positions: evidence of a reading time slowdown in (1b) at either the filled subject position (*Barbara*) or the filled object position (*us*) as compared to the same positions in the declarative sentence in (1a) would suggest that the L2 parser actively posits gaps in licit positions, while a lack of slowdown in the prepositional object position in (2b) as compared to (2a) would suggest avoidance of positing gaps within grammatically unlicensed positions, such as within the Complex Noun Phrase (NP) island (*the boring comments about John's used car*).

(1a) My brother asked if **Barbara** will photograph **us** beside Mom at the graduation.
(1b) My brother asked who **Barbara** will photograph **us** beside ___ at the graduation.
(2a) My sister wondered if the boring comments about **John's** used car were intended to entertain the group.
(2b) My sister wondered who the boring comments about **John's** used car were intended to entertain ___.

The results of a self-paced reading experiment with native speakers of English and Najdi Arabic learners of English indeed showed this pattern: there was a clear reading time slowdown or “filled-gap effect” for both learners and natives at the licit verbal object position (1a, 1b) but not at the prepositional object position within the complex NP island (2a, 2b). In a follow-up study, Canales (2012) revised the stimuli in (2), embedding the critical object within a relative clause island as in (3) so that the critical position in both the licit (1) and illicit (3) contexts followed a verb.

(3a) My brother questioned if the journalist that followed **Henry** last Saturday provoked the guard at the store.
(3b) My brother questioned who the journalist that followed **Henry** last Saturday provoked ________at the store.

Canales (2012) found converging evidence in a study testing Spanish-speaking learners of English, showing evidence of a filled-gap effect at the direct object position (*us*) in (1) but no difference in reading times at the direct object position within the relative clause island (*Henry*) in (3a,b). The presence of the object filled-gap effects across studies suggests that the lack of a reading time slowdown within the island conditions in (2) and (3) is not due to, for example, a lack of statistical power. Both Aldwayan et al. (2010) and Canales (2012) also found limited evidence of subject filled-gap effects (e.g., a reading time slowdown at *Barbara* in 1b as compared to 1a) in both experiments, suggesting that the parser can actively generate a prediction for a gap immediately following the wh-element. While these results were not consistent across experiments or participant groups, both native and learner groups showed evidence of subject filled-gap effects in at least one experiment in each study. The inconsistent emergence of subject filled-gap effects in these studies is not surprising as subject filled-gap effects did not emerge in Stowe's original study, testing English native speakers (see Stowe, 1986; Gibson et al., 1994; Lee, 2004; Johnson, 2015 for further discussion). Overall, the results of the studies discussed above suggest that the L2 parser is guided by syntactic constraints, attempting to resolve wh-dependencies only in licit positions. Using a different paradigm, Omaki and Schulz (2011) and Kim et al. (2015) also provide evidence that Spanish-speaking learners of English actively posit gaps in licit positions but avoid positing gaps in islands. These recent results are in line with several earlier, behavioral studies that showed that L2 learners at very high levels of proficiency are able to show native-like levels of performance on a grammaticality judgment task with respect to the rejection of ungrammatical island violations (e.g., Martohardjono, 1993; White and Juffs, 1998; see review in Belikova and White, 2009).

However, there is a debate as to whether islands are indeed grammatically unlicensed structures and are thus a relevant test case for investigating the recruitment of syntactic knowledge during processing or whether islands are simply processing bottlenecks (e.g., Kluender and Kutas, 1993; Hofmeister and Sag, 2010; Sprouse et al., 2012). It has been proposed that the parser may avoid positing gaps within islands, not due to adherence to syntactic constraints as was suggested above, but because the complex structure inherent to islands simply overwhelms an individual's processing capacities (Kluender and Kutas, 1993; Kluender, 2004; Hofmeister and Sag, 2010).

## Grammatical vs. processing accounts of islands

Under grammatical accounts, gap-filling inside islands is avoided due to constraints on wh-extraction; under these views, both the avoidance of gap-filling in islands during sentence processing and the low acceptability ratings that island-violating sentences incur in acceptability judgment tasks are due to the utilization of syntactic knowledge (e.g., Sprouse et al., 2012). On the other hand, according to recent processing accounts, at least some islands are not the result of grammatical constraints (e.g., Hofmeister and Sag, 2010). Instead, the appearance of island sensitivity during processing and the elicitation of low ratings for island-violating sentences in judgment tasks are a consequence of processing pressures which are argued to increase difficulty in resolving wh-dependencies. On these accounts, island effects emerge when various processing burdens combine to render processing particularly difficult, leaving few resources to resolve dependencies. These processing burdens arise from a range of factors that are not unique to islands. They include the presence of the filler-gap dependency itself, which is argued to incur a processing cost that may increase as distance increases, the processing of additional referents along the path between the filler and gap, processing clause boundaries, and how complex or semantically rich the filler phrase is, among other factors (e.g., Kluender, 1992, 1998, 2004; Hofmeister and Sag, 2010; Hofmeister et al., 2013; see also Cinque, 1990). Since these factors are hypothesized to lead to island effects, manipulating them in order to ease processing difficulty is expected to ameliorate or remove island effects. This expectation is arguably not shared by grammatical accounts, which hold that the parser should not predict a gap within an island context, regardless of such factors. In support of the processing accounts, several studies have put forth evidence that manipulating one or more non-structural factors leads to both improved judgments and reduced processing difficulty as indexed by self-paced reading times. For example, Hofmeister and Sag (2010, Experiment 2) used self-paced reading to investigate whether the processing difficulty and low acceptability ratings of wh-islands such as (4b) below would be ameliorated by simply replacing a bare wh-element (*Who*) with a more complex, semantically rich wh-phrase (*Which employee*). Participants first read a lead-in sentence like (4a) below, and then a second sentence with either a bare wh-phrase in a wh-island construction (4b), a which phrase in a wh-island construction (4c), or a baseline condition involving no island violation (4d).

(4a) Albert learned that the managers dismissed the employee with poor sales after the annual performance review.
(4b) Bare Wh-phrase: Who did Albert learn whether they dismissed after the annual performance review?
(4c) Which phrase: Which employee did Albert learn whether they dismissed after the annual performance review?
(4d) Grammatical Baseline: Who did Albert learn that they dismissed after the annual performance review?

Reading times at the three regions following the embedded verb (*dismissed* in 4 above) were significantly faster for the Which phrase condition (4c) than the Bare Wh-phrase condition (4b); indeed, reading times for the Which phrase condition did not differ from the grammatical baseline condition (4d). These results suggest that manipulation of the semantic complexity of the filler phrase may reduce the difficulty of processing the wh-island. In a follow-up where bare wh- and which phrase sentences (presented in embedded questions) were rated for acceptability, the which-phrase sentences received higher acceptability ratings. That the manipulation of this factor both eased processing difficulty following the gap site and improved acceptability ratings was taken to suggest that processing pressures contribute to island effects.

Individual differences in processing resources (in particular, working memory) constitute another factor that may affect whether island effects emerge (e.g., Kluender, 1992; Hofmeister et al., 2012a,b). As Hofmeister and Sag (2010) point out, “Notably, some individuals seem fairly accepting of island violations, while others reject the same tokens. This type of variation in acceptability judgments, both within and across subjects emerges naturally on the processing account of islands. Individuals are known to differ significantly from one another in terms of working memory capacity (Daneman and Carpenter, 1980; King and Just, 1991; Just and Carpenter, 1992), and the same individual may have more or fewer resources available, depending upon factors such as fatigue, distractions, or other concurrent tasks” (p. 403). However, in cases of extreme processing difficulty, such individual differences may not emerge (e.g., Hofmeister et al., 2014).

In a series of large-scales studies, Sprouse et al. (2012) examined whether individual differences in processing resources modulate the acceptability of islands using off-line acceptability rating tasks and two measures of working memory capacity. The acceptability judgment tasks, testing four types of islands, used a factorial design manipulating the presence or absence of an island structure, as well as the position of the gap (in the matrix clause or in the embedded clause) as in (5). Sprouse et al. examined whether the combined effect of the presence of an island structure and extraction as in (5d) was “superadditive,” yielding lower acceptability ratings than would be expected by the addition of the two individual factors.

(5a) Who ____ claimed that John bought the car?
          non-island/matrix
(5b) What did you claim that John bought ____?
          non-island/embedded
(5c) Who ___ made the claim that John bought a car?
          island/matrix
(5d) ^*^What did you make the claim that John bought ____?
          island/embedded

According to Sprouse et al. (2012), processing accounts should further predict that effects of superadditivity or sensitivity to island violations would be reduced in those with superior processing resources. Grammatical accounts predict no such relationship. Sprouse et al. (2012) argue that their results showed no meaningful relationship between working memory and the “superadditive” effect on acceptability judgments that they observed, taking these findings to support the grammatical accounts of islands.

However, in response, Hofmeister et al. (2012a,b) point out that the lack of a relationship between acceptability ratings and processing resources in Sprouse et al. (2012) could be due to the nature of the tasks used. Hofmeister et al. argue that the stimuli tested, which included decontextualized questions with bare wh-fillers may have been particularly difficult to process, not allowing the variability in acceptability judgments that would allow a correlation to emerge. They also claim that the working memory tasks (n-back and serial recall) used in Sprouse et al. (2012) may assess short term memory, as opposed to working memory (see also Conway et al., 2005), and have not been shown to capture variability in sentence processing in other contexts.

Aldosari (2015) modified Sprouse et al.'s stimuli in order to address some of the concerns raised by Hofmeister et al. (2012a,b). In the acceptability judgment task, Aldosari included a context sentence which preceded the wh-question so as to not present decontextualized questions. The wh-questions themselves were revised to include lexical wh-fillers as opposed to bare wh-words (Hofmeister and Sag, 2010; see also Goodall, 2015). The goal of these revisions was to potentially decrease the processing difficulty in order to allow more room for variability to emerge in the judgments. Finally, a complex span task (operation span) was used. The results for both native speakers and Najdi Arabic learners of English showed, in line with Sprouse et al., clear effects of superadditivity, with low acceptance of the island sentences. In addition, the results revealed no significant relationship between working memory and sensitivity to island violations for either native speakers or L2 learners.

In addition to these recent studies examining the relationship between processing abilities and acceptability judgments, previous studies have also utilized reading-time measures in order to test whether islands can be indeed be explained via processing limitations, without recourse to grammatical constraints. The approach in these studies (e.g., Phillips, 2006; Wagers and Phillips, 2009) has been to examine whether gaps are posited in linguistic contexts that typically constitute islands, but can under some circumstances be rescued by later material in the sentence. For example, extraction from complex subjects (e.g., 6a below) is typically prohibited; however, extraction from subject islands is acceptable in “parasitic gap” constructions in which the wh-element is associated with two different gaps, one within the subject island and a second, object gap, which can “rescue” the violation (as is shown in 6b). Note, however, that a second gap cannot rescue the first if the verb is finite, as in (6c).

(6a) ^*^What did [the plan to build *t*] ultimately destroy the house?
(6b) What did [the plan to build *t*] ultimately destroy *t* ?
(6c) ^*^What did [the group that built *t*] ultimately destroy *t* ?

Phillips (2006) reasoned that, if the subject island in (6) results from processing pressures which simply make it too difficult to resolve the dependency there, then the possibility that extraction from that position may be rescued by the presence of a subsequent gap (as is true in non-finite structures like 6b) should not matter; a gap should never be posited in that position.

Phillips (2006) provided reading-time evidence that a gap was indeed posited within a subject island when the structure was non-finite, and thus potentially rescuable by a subsequent gap, but not when the structure was finite (see Ross, 1967). These results were taken to be consistent with grammatical accounts of islands. However, Hofmeister and colleagues challenge this interpretation, pointing out that, under their view, islands are positions that are difficult rather than impossible to extract from, and that factors like verb finiteness may indeed modulate how difficult it is to process the clause, thus rendering gap filling within the subject island more vs. less likely (Kluender, 2004; Hofmeister et al., 2013).

Using eye-tracking, Boxell and Felser (2013) replicated the results of Phillips (2006) with a group of native speakers but showed a somewhat different pattern for native German learners of English. According to first-pass reading measures, while native speakers posited gaps in islands only when such gaps might ultimately be rescued, L2 learners initially posited gaps in islands across the board (see also Kim et al., 2015). The L2 learners did however show a native-like pattern at the critical region in rereading time, a measure that includes all fixations in a region after it has been exited.

The distinct pattern that emerges in the early reading measures for the native speakers and L2 learners leads Boxell and Felser (2013) to propose that L2 processing differs significantly from native processing: while native speakers are immediately constrained by island restrictions, L2 learners' sensitivity to island constraints is delayed. A recent study by Felser et al. (2012) also argues that native and non-native processing differ in terms of the type of information that is prioritized at different stages of processing. Felser et al. (2012) conducted two experiments, one with a filled-gap paradigm and the other with a plausibility mismatch paradigm, both examining whether learners and natives would attempt to resolve wh-dependencies in non-island contexts but avoid positing gaps in relative clause islands. In the filled-gap experiment, a filled-gap effect emerged for natives at the critical region and for learners at the spillover region. Neither group attempted to resolve wh-dependencies in islands. In the plausibility mismatch experiment, it was the L2 learners who showed an immediate plausibility mismatch effect only for the non-island structures; the same effect for natives emerged in re-reading measures, also at the critical region. The results of the Boxell and Felser (2013) and Felser et al. (2012) study differ critically in that the learners in the Felser et al. study do not attempt to resolve wh-dependencies in islands at any point while the results of Boxell and Felser (2013) suggest an initial insensitivity to islands. Boxell and Felser speculate that differences in the processing complexity of the two different types of islands (subject islands vs. relative clause islands) may account for the differences in the two studies. The present study will further address whether L2 learners demonstrate island sensitivity similarly to native speakers; in addition, we bring together two strands of research discussed above by examining whether there is a relationship between individual differences and the online processing of wh-dependencies in both island and non-island contexts.

## Present study

In the current study, we examine the relationship between working memory and filled-gap effects in both native speakers and L2 learners^1^. To our knowledge, no previous study has directly examined the relationship between processing abilities and filled-gap effects in islands, which provide an online measure of the processing of wh-dependencies. However, this approach may be advantageous as it is possible that offline measures of acceptability do not capture variability that may emerge in the course of processing the island itself. Grammatical accounts do not predict a relationship between working memory and the establishment of wh-dependencies in island contexts as the parser should simply not attempt to resolve dependencies in grammatically unlicensed positions. In contrast, a processing account such as that proposed by Hofmeister and colleagues predicts that working memory and island sensitivity may be related (e.g., Hofmeister and Sag, 2010). Thus, as suggested by Sprouse et al. (2012), results showing that individuals with better processing abilities are better able to establish wh-dependencies in complex structures such as islands would be consistent with this kind of account, a claim that Hofmeister et al. (2012b) acknowledge to be broadly in line with their proposal.

On the other hand, finding a relationship between working memory and the processing of grammatically licensed wh-dependencies would be consistent with both proposals. One possibility is that lower working memory may lead to greater filled-gap effects in licit positions. A number of models highlight effects of distance on dependency resolution, pointing out that the resolution of wh-dependencies becomes more difficult at a greater distance (e.g., Gibson, 1998) although the reasons for these effects and the specific circumstances under which increased distance indeed engenders processing burden remain a matter of investigation (e.g., Wagers and Phillips, 2014; Nicenboim et al., 2015). Considering that wh-dependency resolution may generally become more burdensome as distance increases (thus leading the parser to resolve the dependency as soon as possible; e.g., Frazier, 1987), perhaps those participants with low working memory will show greater eagerness to quickly resolve the dependency, and thus yield greater evidence of active gap-filling than those with high working memory.

However, there is also reason to speculate that higher working memory would lead to greater filled gap-effects in licit positions. Resolving wh-dependencies involves a range of processes, from initially encoding the wh-dependency, which is argued to involve generating predictions for upcoming gap sites in advance of unambiguous bottom-up evidence (e.g., Nakano et al., 2002; Lee, 2004; Omaki et al., 2015), to maintaining and/or retrieving dependency-related information while also processing bottom-up information, monitoring for conflicts among expected and encountered material, and ultimately resolving the dependency. All of these processes have been argued to make recourse to working memory or other resources related to attentional control (e.g., Daneman and Carpenter, 1983; Engle, 2002; Hutchison, 2007; Slevc and Novick, 2013). It may thus be those with greater resources who are more likely to successfully engage these processes.

Some evidence suggesting that higher working memory may lead to greater gap-filling effects comes from Nakano et al. (2002), who examined pre-verbal gap filling in Japanese using the cross-modal lexical priming paradigm. Nakano et al. examined whether evidence for pre-verbal gap filling depended on working memory, finding that only those participants with high working memory showed evidence of pre-verbal trace reactivation (see also Roberts et al., 2007). While there remains a paucity of studies directly examining individual differences in working memory/attentional control in wh-dependency resolution (Nicenboim et al., 2015), the above evidence is consistent with the prediction that those with higher working memory may show greater filled-gap effects in licit positions.

Examples of the target stimuli in our experiment, which were adapted from Canales (2012), are given below in (7) and (8). Our first comparison involves sentences that do not contain an island structure, the *Non-Island* sentences in (7a-b). The comparison of reading times for *Non-Island* sentences that do (7a) and do not (7b) involve wh-extraction allows us to probe for filled-gap effects in licit, filled subject (*Chris*) and filled object (*Tom*) positions. Our second comparison involves sentences that contain a relative clause island, the *Island* sentences in (8a,b). The comparison of *Island* sentences that do (8a) and do not (8b) involve wh-extraction allows us to probe for filled-gap effects both in the licit filled subject position (*the actress*) and a filled object position within the relative clause island (*Tyler*).

*Non-Island*, No extraction
  (7a) The instructor wondered if **Chris** will film **Tom** with Susan at the reception.
  *Non-Island*, Wh-extraction
(7b) The instructor wondered who **Chris** will film **Tom** with ____ at the reception.
  *Island*, No extraction
(8a) My father asked if **the** actress that married **Tyler** last summer kissed the director during_15_ the_16_ rehearsal_17_.
  *Island*, Wh-extraction
(8b) My father asked who **the** actress that married **Tyler** last summer kissed ____ during the rehearsal.

The present study examines both native speakers and native Korean learners of English in order to better understand the nature of the processing of wh-dependencies in both native and learner populations. Previous studies have shown that Korean learners may not abide by island constraints during online processing (Kim et al., 2015), which they suggest may be due to the fact that Korean is a wh-*in situ* language which does not exhibit overt wh-movement (Sohn, 1980, 1999). However, as Kim et al. (2015) acknowledge, some recent papers have suggested that wh-*in situ* languages, such as Korean, do block extraction from relative clauses, just as in English (Han and Kim, 2004; Phillips, 2013). Our previous work with Najdi Arabic learners of English has also shown that is possible for native speakers of a wh-*in situ* language to abide by island constraints during processing. Thus, we include both native speakers and Korean learners of English to compare native and non-native processing broadly, but not necessarily to examine potential effects of L1 transfer.

The present study examines whether native speakers and learners show qualitatively similar patterns, as has been shown in some studies (e.g., Aldwayan et al., 2010; Omaki and Schulz, 2011), or whether learners are unable to use syntactic information on the same timecourse as native speakers (Felser et al., 2012; Boxell and Felser, 2013). If the two groups show qualitatively similar patterns, a filled-gap effect should emerge at the grammatically licensed direct object position in the *Non-Island* sentences (*Tom* in 7b) but not within the relative clause island in the *Island* sentences (*Tyler* in 8b) for both groups. In contrast, if learners are unable to prioritize syntactic information and use it in the earliest stages of processing (Felser et al., 2012), then learners should either show filled-gap effects at the direct object positions in both the *Non-Island* and *Island* sentences, suggesting an attempt to resolve wh-dependencies within islands (Boxell and Felser, 2013; Kim et al., 2015) or they should show sensitivity to island contexts only at a delay. With respect to the second possibility, learners may, for example, pattern similarly to native speakers, positing a gap in (7b) and avoiding positing a gap within the island (no difference between 8b) but this pattern should emerge on a different timecourse from native speakers, perhaps emerging at a region later in the sentence as has been observed in previous studies (e.g., Felser et al., 2012). We will also examine effects at the licit filled subject positions in both Non-Island and Island sentences (*Chris* in 7b; *the actress* in 8b). However, as discussed above, the inconsistency of subject filled-gap effects in both native speakers and L2 learners in previous experiments using this same design (Stowe, 1986; Lee, 2004; Aldwayan et al., 2010; Canales, 2012) does not allow us to make strong predictions regarding similarities and differences between learners and native speakers. We will return to this issue in the discussion.

The study also examines the nature of islands, investigating whether there is a relationship between working memory and the processing of wh-dependencies in islands. No such relationship is predicted by the grammatical accounts. A positive relationship between working memory and the size of the filled-gap effect in the object position within the relative clause island (*Tyler* in 8a,b) would be consistent with Hofmeister and colleagues' versions of the processing account (e.g., Hofmeister et al. 2012a,b, 2014). Any significant relationships that emerge between working memory and the grammatically licensed potential gap sites (subject positions in 7b and 8b, object position in 7b) would be consistent with both proposals^2^.

## Materials and methods

### Participants

Forty-nine advanced Korean learners of English and 54 native English speakers participated in the study. The Korean learners (mean age = 28.41; 28 females) were recruited from the University of Kansas and its surrounding community; their mean age of arrival was 22.89 years old. All learners reported no significant exposure to English before age of 12, and no learner reported significant exposure to any wh-movement language other than English. The learners' English proficiency was assessed using the University of Michigan Listening Comprehension Test, a 45 question test which covers various aspects of English grammar (mean proficiency score = 39.39). Eight additional Korean learners and eight additional native English speakers also participated in the study, but were identified as outliers with respect to magnitude of their filled-gap effects and excluded from the final analysis of the data, and one additional English speaker also participated but was excluded from the final analysis for showing exceptionally fast reading times (faster than 250 ms) across regions, as described in the *Data Analysis* section below. The Korean learners of English were provided with payment for their participation, and the native English speakers (mean age = 21.15; 41 females), who were all students at the University of Kansas, completed the study for extra credit. This study was approved by the Institutional Review Board of the University of Kansas and all participants provided their written informed consent before participating.

### Stimuli

#### Non-island stimuli

The *Non-Island* stimuli included 20 pairs of sentences, with each pair consisting of a control sentence with no extraction (9a) and a matched wh-extraction sentence (9b); the region number for each word is indicated by the subscripts in (9). A full list of stimuli is provided as Supplementary Material.

*Non-Island*, No extraction
(9a) The_1_ instructor_2_ wondered_3_ if_4_ **Chris**_5_ will_6_ film_7_ **Tom**_8_ with_9_ Susan_10_ at_11_ the_12_ reception_13_.
  *Non-Island*, Wh-extraction
(9b) The_1_ instructor_2_ wondered_3_ who_4_ **Chris**_5_ will_6_ film_7_ **Tom**_8_ with_9_ _____10_ at_11_ the_12_ reception_13_.

The wh-structure in (9b) involves extraction from the grammatically licit prepositional object position (region 10). Preceding this position are two grammatically licit potential gap positions that are filled with lexical material: the embedded subject position (region 5) which is filled with the subject *Chris*, and the post-verbal direct object position (region 8) which is filled with the object *Tom*; these positions are bolded in example (9) above. These two regions and their spillover regions (region 6 and 9, respectively) serve as critical regions to test for filled-gap effects in positions from which wh-extraction is grammatically licit.

The embedded verbs used in region 7 were all transitive verbs. The prepositional objects (region 8) were all proper names that were three letters long, and the embedded subjects (region 5) were all proper names as well (mean length = 5.4 letters, range 4–11 letters).

#### Island stimuli

The *Island* stimuli included 20 additional pairs of sentences, with each pair consisting of a control sentence with no extraction (10a) and a matched wh-extraction sentence (10b).

*Island*, No extraction
(10a) My_1_ father_2_ asked_3_ if_4_ **the**_5_ actress_6_ that_7_ married_8_ **Tyler**_9_ last_10_ summer_11_ kissed_12_ the_13_ director_14_ during_15_ the_16_ rehearsal_17_.
  *Island*, Wh-extraction
(10b) My_1_ father_2_ asked_3_ who_4_ **the**_5_ actress_6_ that_7_ married_8_ **Tyler**_9_ last_10_ summer_11_ kissed_12_ _____13−14_ during_15_ the_16_ rehearsal_17_.

The wh-structure in (10b) involves extraction from the grammatically licit object position (regions 13–14). Crucially, preceding this position is a relative clause island, from which wh-extraction is illicit. While the relative island contains a post-verbal object position (region 9) filled with a proper name (*Tyler*, in 10b above), extraction from this position is not grammatically licensed. Thus, region 9 and its spillover region (region 10) serve as critical regions to probe for filled-gap effects in a grammatically illicit position (within a relative clause island). Region 5 and its spillover region (region 6) constitute the filled embedded subject position, a grammatically licit site for extraction. Like the embedded subject position in the *Non-Island* sentences, the embedded subject region 5 and its spillover region (region 6) serve as critical regions to test for filled-gap effects in the grammatically licit, subject position.

The verbs inside the relative clause island (region 8) were all transitive verbs. The post-verbal object position within the island (region 9) was always filled with a proper name that was five letters long. An adverbial phrase (e.g., *last summer*) always followed region 9 (e.g., *Tyler*) in order to provide a spillover region following the post-verbal object position that would precede the verb that licenses the actual gap position in the wh-extraction sentence (e.g., *kissed*). The embedded subject position from which extraction is grammatically licensed (region 5 and its spillover region, region 6) were comprised of a determiner-noun combination; the determiner in region 5 was always the three-letter-long determiner “the.”

The 20 the *Non*-*Island* sentences, the 20 *Island* sentences, and 80 filler sentences were presented together, yielding a 1:2 target-to-filler ratio. Two Latin-square lists were created, such that every participant was presented with either the extraction or no-extraction version of every sentence, but no participant read more than one version of a given sentence. The sentences were presented in different randomized order for each participant.

### Procedure

All participants completed a background questionnaire, the self-paced reading task, and then two working memory tasks (the reading span task and the counting span task) which are described below; the order of the two working memory tasks was counterbalanced across participants. Korean learners of English also completed the University of Michigan Listening Comprehension Test (1972), after completing all other tasks. The self-paced reading task, working memory tasks and proficiency test were all administered using *Paradigm* presentation software (Tagliaferri, 2005).

#### Self-paced reading task

Each sentence was presented word-by-word in a non-cumulative moving window self-paced reading paradigm (Just et al., 1982). At the beginning of each trial, each word of the sentence was masked by a series of dashes; this masking included words and punctuation, but did not include the spaces between words. Each time the participant clicked a mouse button to advance through the sentence, the next word was unmasked, and the previous word was masked again. After the last word of each sentence, the sentence was then presented again in full, but with one word missing (e.g., “My _____ asked if the actress that married Tyler last summer kissed the director during the rehearsal.”). Participants selected the missing word from among two options (e.g., “father” and “sister”) which were presented on the screen, by pressing the appropriate key on the computer keyboard (either the key labeled “L” for the word on the left of the screen, or that labeled “R” for the word on the right of the screen). Prior to the experiment, participants completed a practice session consisting of five practice sentences. Participants were instructed to read the sentences naturally for comprehension, and to answer the end-of-sentence question as accurately as possible. Breaks were provided after 40 and 80 trials.

#### Working memory tasks

Participants completed a verbal measure of working memory, the reading span task (Daneman and Carpenter, 1980), and a non-verbal measure of working memory, the counting span task (Case et al., 1982). These tasks are argued to reflect working memory rather than short-term memory, as they involve both a memory component and a processing component, which interferes with rehearsal. Both tasks were presented to the native English speakers and the Korean learners of English in their native language, as it has been argued that measures of working memory capacity which are given in the second language are affected by the second language learners' English proficiency (e.g., Harrington and Sawyer, 1992; Juffs and Harrington, 2011).

In the reading span task, following the protocol in Conway et al. (2005), participants were asked to read sentences out loud and make sensicality judgments, while remembering random letters of the alphabet which followed each sentence (Kim, 2008). On each trial, the participant read the sentence out loud into a microphone, provided the sensicality judgment, and then said the letter that followed the sentence out loud, which triggered the next sentence in the series to immediately appear. After a series of 2–5 sentences, the participant was shown a screen prompting them to enter the letters that followed the previous set of sentences. Participants entered the recalled letters into boxes on this screen and were instructed to use a period (.) as a placeholder for letters that they could not recall.

The counting span task required participants to count target visual stimuli mixed in with distractor stimuli in a series of successive displays, while remembering the total number of target stimuli for each individual display (Conway et al., 2005). In each trial, the participant was presented with an array of target objects (dark blue circles) and distractor objects (light green circles); upon presentation of this array, the participant counted the number of target stimuli out loud, repeating the total, at which point the experimenter immediately entered the total using a computer keyboard, which triggered the next trial to begin. After a series of 2–6 trials, the participant was shown a screen prompting them to enter the total number of target objects from each of the previous arrays they had been presented. Participants entered the totals that they recalled into boxes on this screen, and entered a period (.) as a placeholder for any totals that they could not recall.

For both the reading span task and the counting span task, participants were instructed to respond as quickly and accurately as possible. Stimuli within each task were presented in a randomized order. The entire testing session, including all of the above-mentioned tasks, took ~60 min for native English speakers and 75 min for the Korean learners of English.

### Data analysis

As mentioned in the *Participants* section above, in addition to the 49 advanced Korean learners of English (mean age = 28.41; range 18–48 years old) and 54 native English speakers (mean age = 21.09; range 17–65 years old) reported in the current study, eight additional Korean learners of English and eight native English speakers were initially tested but identified as outliers and excluded from the final analysis, since their filled-gap effects were >3 standard deviations from the mean effect size of the dataset as a whole. Using filled-gap effect size as a value for identifying outliers is motivated by the fact that filled-gap effect size is a primary variable of interest in the regression analyses reported below. While these outliers are of most concern for the regression analyses, in order to keep the participant groups identical in the ANOVA analyses reported below (which probe for the presence of filled-gap effects in grammatically licit positions and for the avoidance of gap-filling inside islands) and in the regression analyses (which examine the relationships between individuals' filled-gap effect size and working memory), these participants were removed from both types of analysis. One additional native English speaker was also removed prior to analysis as this participant read at an extremely fast rate (faster than 250 ms) across regions and conditions.

For the dataset reported here, overall mean accuracy rate for the end-of-sentence question was 96.3% for native speakers and 93.4% for Korean learners of English; no participant in either group performed at < 80% accuracy. Only those trials for which the end-of-sentence question was answered correctly were carried forward for statistical analysis. For *Non-Island* sentences, this resulted in exclusion of 3.43% of the data for native English speakers and 6.43% of the data for the Korean learners of English. For *Island* sentences, this resulted in exclusion of 3.8% of the data for the native English speakers and 6.73% of the data for the Korean learners of English.

Residual reading times were calculated by subtracting the raw reading time from the reading time predicted given a word's length by a regression equation that was constructed separately for each participant. Residual reading times beyond 2 standard deviations from the participant's mean for a given condition in a given region were excluded from the analysis (Ratcliff, 1993). For *Non-Island* sentences, this resulted in exclusion of 3.88% of the data for the native English speakers and 3.85% of the data for the Korean learners of English. For *Island* sentences, this resulted in exclusion of 4.1% of the data for the native English speakers and 4.03% of the data for the Korean learners of English.

2 × 2 mixed repeated-measures ANOVAs were performed on the remaining data, both by participants (*F*_1_) and by items (*F*_2_). For both the *Non-island* and the *Island* comparisons, the between-subjects factor was Group (native vs. learner) and the within-subjects factor was Condition (wh-extraction vs. no extraction). The critical regions for *Non-Island* sentences were region 8 (object filled-gap) and its spillover region (region 9), as well as region 5 (subject filled-gap) and its spillover region (region 6). The critical regions for *Island* sentences were region 9 (illicit object filled-gap within the relative clause island) and its spillover region (region 10), as well as region 5 (subject filled-gap) and its spillover region (region 6).

We also conducted a regression analysis to examine the relationship between filled-gap effect size and working memory both in grammatically licit positions and inside islands. For this analysis, we calculated for each individual the difference in mean reading times between the no extraction and wh-extraction conditions (subtracting the no extraction from the wh-extraction condition) in a given critical region; this measure, which we refer to throughout as Filled-gap Effect Size, serves as the dependent variable for the regression analyses. In order to obtain an independent variable reflecting working memory, we averaged for each individual their scores on the reading span task and the counting span task to create a Combined Working Memory Score. We use this score rather than the separate scores for each of the two working memory measures because the scores on these two measures are highly correlated (*r* = 0.528, *p* < 0.001). Because cognitive functioning, which includes working memory, declines with age (e.g., Hess, 2005; Oberauer, 2005; McArdle et al., 2007; Nettelbeck and Burns, 2010; Wass et al., 2012), we also control for age in our regression models. For both the ANOVA analyses and the regression analyses, we interpret *p* < 0.05 as significant and *p-*values between 0.05 and 0.10 as marginal.

Working memory score was calculated as a score from 1 to 100 based on percent of letters (for the reading span task) or numbers (for the counting span task) that were accurately recalled. Korean learners of English scored an average of 61.95% (range of 33.46–93.75%) on this composite measure of working memory, as compared to 59.73% (range of 29.25–88.93%) for native speakers of English. We used partial-unit scoring such that participants were given credit for each letter or number recalled in the correct position within a given trial. Performance on the processing tasks was not included in the working memory score, following the protocol outlined in Conway et al. (2005), who discuss the fact that accuracy on the processing tasks often correlates with the recall accuracy of the target items^3^.

To address whether higher working memory capacity facilitates gap filling within or outside islands, we completed a sequential regression analysis for each critical and spillover region, while controlling for the effects of age. Filled-gap Effect Size at each region was regressed on age, centered scores of Combined Working Memory, and Group (native = 0, L2 learner = 1) in the first block of a sequential regression. The cross-product of the centered Combined Working Memory scores and Group was then added in the second block and Δ*R*^2^ was examined to determine if an interaction between groups was present. Follow-up analyses were conducted for those regions showing an interaction.

## Results

### Filled-gap effects

#### Object filled-gap effects

**Figure 1:**
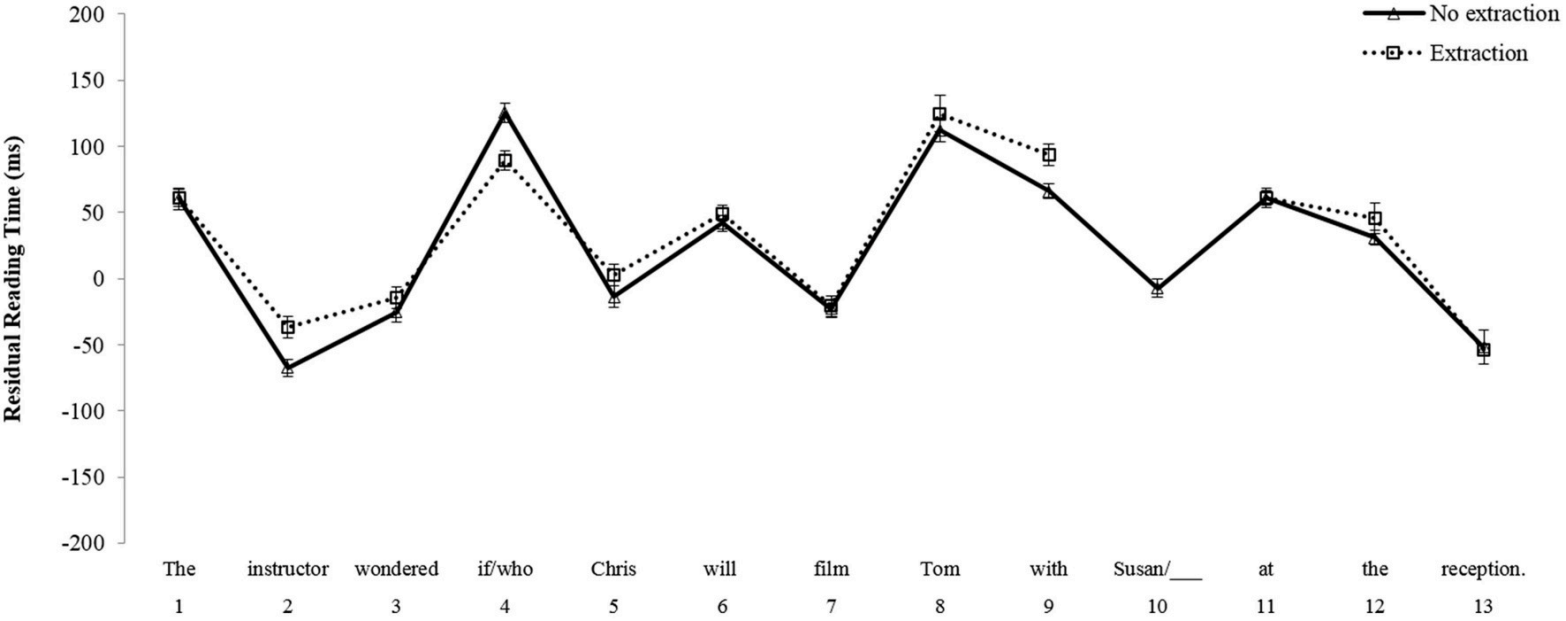
**Mean reading times by participants for Non-Island sentences, Native English speakers**.

**Figure 2:**
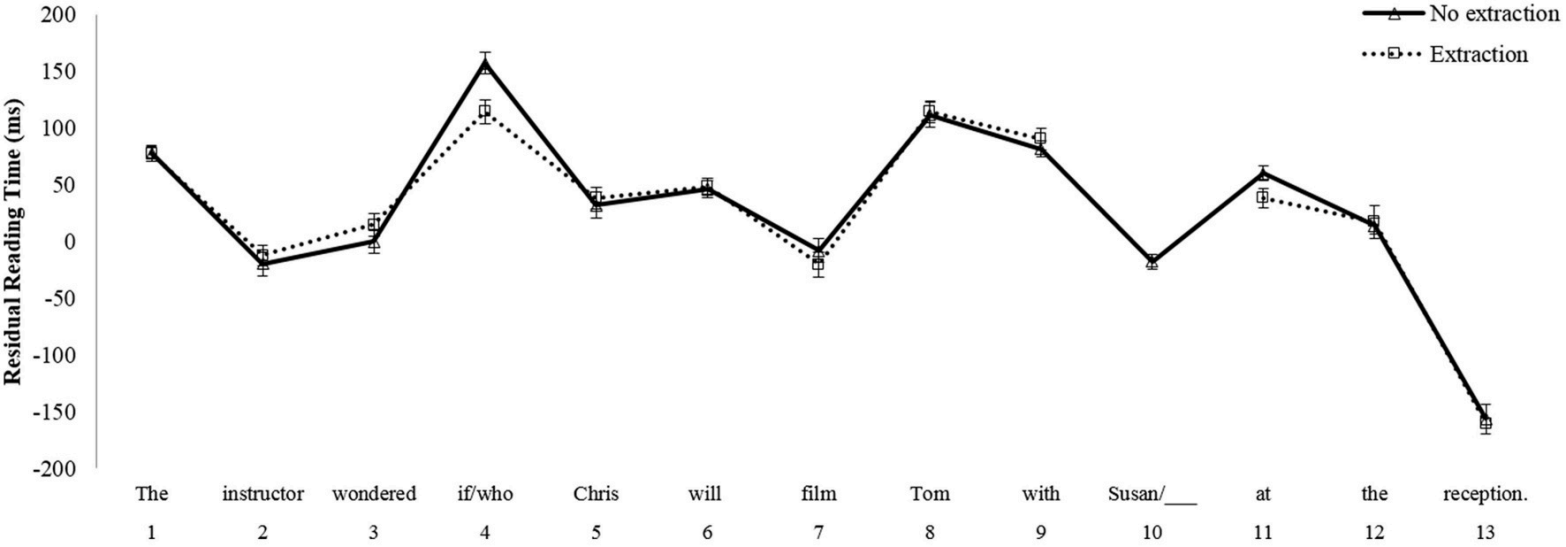
**Mean reading times by participants for Non-Island sentences, Korean learners of English**.

In the *Non-Island* comparison, the results of the mixed repeated measures ANOVA for region 8, the critical post-verbal object position from which extraction is grammatically licit, did not reveal main effects of Group [*F*_1(1, 101)_ = 0.18, *p* = 0.67; *F*_2(1, 38)_ = 0.004, *p* = 0.95] or Condition [*F*_1(1, 101)_ = 0.802, *p* = 0.372; *F*_2(1, 38)_ = 2.160, *p* = 0.15]. Furthermore, there was no interaction between these factors [*F*_1(1, 101)_ = 0.308, *p* = 0.58; *F*_2(1, 38)_ = 2.750, *p* = 0.11]. However, a main effect of Condition emerged at region 9 [*F*_1(1, 101)_ = 6.032, *p* < 0.05; *F*_2(1, 38)_ = 13.967, *p* < 0.01], reflecting a reading time slowdown in the wh-extraction condition as compared to the no extraction condition. There was no main effect of Group [*F*_1(1, 101)_ = 0.657, *p* = 0.419; *F*_2(1, 38)_ = 1.909, *p* = 0.18] nor was there an interaction at region 9 between Group and Condition [*F*_1(1, 101)_ = 1.609, *p* = 0.207; *F*_2(1, 38)_ = 0.160, *p* = 0.69]. Mean reading times for native English speakers in the *Non-Island* sentences are shown in Figure 1, and those for Korean learners of English are shown in Figure 2.

**Figure 3:**
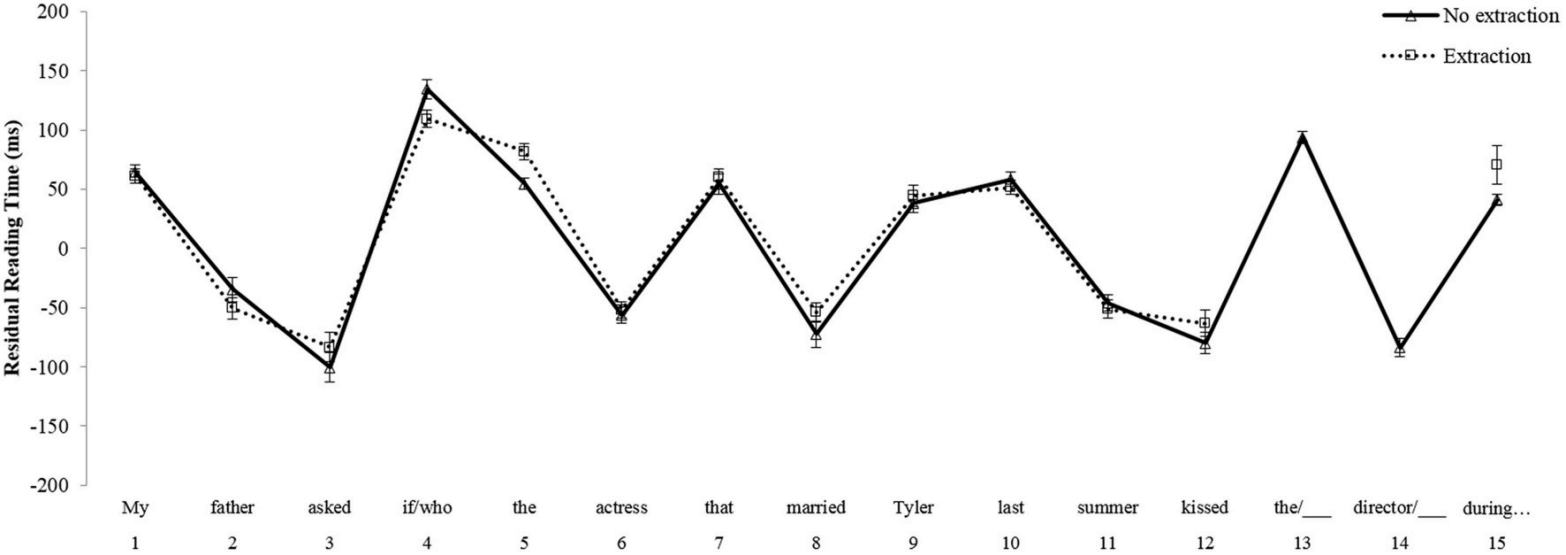
**Mean reading times by participants for Island sentences, Native English speakers**.

**Figure 4:**
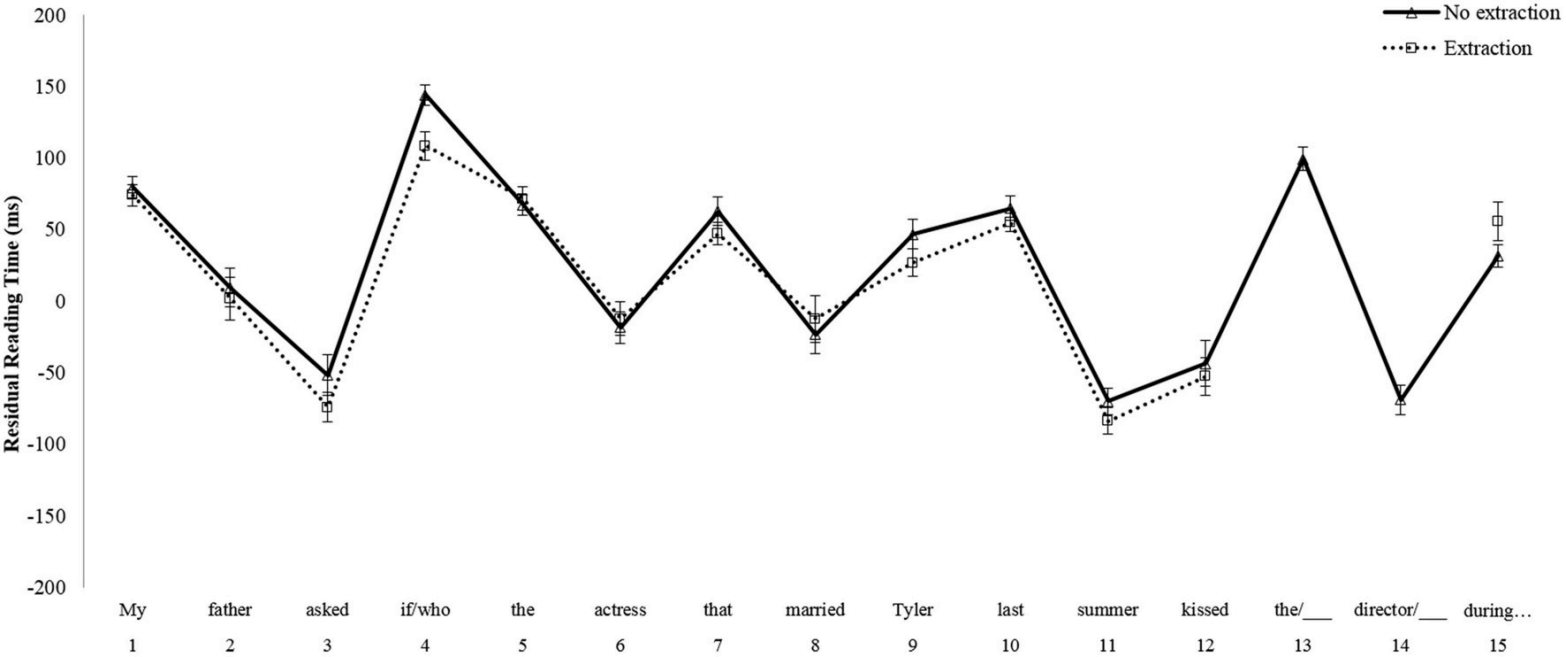
**Mean reading times by participants for Island sentences, Korean learners of English**.

For the *Island* comparison, no main effects of Group [*F*_1(1, 101)_ = 0.208, *p* = 0.65; *F*_2(1, 38)_ = 1.479, *p* = 0.23] or Condition [*F*_1(1, 101)_ = 0.779, *p* = 0.38; *F*_2(1, 38)_ = 1.269, *p* = 0.27] emerged at the critical region 9, the post-verbal object position within the relative clause island. There was a marginal Group by Condition interaction at region 9 in the by-participants analysis [*F*_1(1, 101)_ = 3.008, *p* = 0.09; *F*_2(1, 38)_ = 2.177, *p* = 0.148]. However, *post-hoc t*-tests revealed that the reading time difference between the wh-extraction condition and the no extraction condition was not significant for either native English speakers [*t*_(53)_ = –0.729, *p* = 0.47, two-tailed paired *t-*test] or Korean learners of English [*t*_(48)_ = 1.578, *p* = 0.12, two-tailed paired *t-*test]. At the spillover region, region 10, there was a main effect of Group in the by-items analysis only [*F*_1(1, 101)_ = 0.365, *p* = 0.55; *F*_2(1, 38)_ = 4.173, *p* < 0.05]. This effect reflected the fact that residual reading times were slower overall for Korean learners of English than for native English speakers. Additionally, there was an effect of Condition in region 10 which reached significance only in the by-items analysis [*F*_1(1, 101)_ = 2.605, *p* = 0.11; *F*_2(1, 38)_ = 5.240, *p* < 0.05]. However, this effect was in the opposite direction of what would be expected if a filled-gap effect were to emerge; participants read faster in the wh-extraction condition as compared to the no extraction condition. There was also no interaction between Group and Condition at region 10 [*F*_1(1, 101)_ = 0.096, *p* = 0.76; *F*_2(1, 38)_ = 0.484, *p* = 0.49]. Overall, the results from the *Non-Island* comparison indicate that, although numerically small, a significant filled-gap effect emerged for both groups at the spillover region of the filled direct object position. In contrast, as evidenced by the results from the *Island* comparison, neither native English speakers, nor Korean learners of English show a filled-gap effect within the relative clause island. Mean reading times for the *Island* sentences for native English speakers are illustrated in Figure 3, and those for Korean learners of English are shown in Figure 4.

#### Subject filled-gap effects

In addition to examining whether native English speakers and Korean learners of English showed evidence of object filled-gap effects, we also examined the critical region 5 and spillover region 6 in both the *Non-Island* and the *Island* sentences for possible subject filled-gap effects. Recall that for both sentence types, the subject gap positions are licit positions for wh-extraction.

In the *Non-Island* comparison, there was no effect of Condition in either region 5 [*F*_1(1, 101)_ = 1.612, *p* = 0.21; *F*_2(1, 38)_ = 2.523, *p* = 0.12] or region 6 [*F*_1(1, 101)_ = 0.926, *p* = 0.34; *F*_2(1, 38)_ = 2.082, *p* = 0.16]. There was an effect of Group in region 5 [*F*_1(1, 101)_ = 17.157, *p* < 0.001; *F*_2(1, 38)_ = 4.990, *p* < 0.05], reflecting the fact that Korean learners of English yielded slower residual reading times overall compared to native English speakers. There was no effect of Group in region 6 [*F*_1(1, 101)_ = 0.05, *p* = 0.82; *F*_2(1, 38)_ = 0.010, *p* = 0.92]. There was no interaction between Group and Condition in either the critical region 5 [*F*_1(1, 101)_ = 0.309, *p* = 0.579; *F*_2(1, 38)_ = 0.069, *p* = 0.79] or the spillover region 6 [*F*_1(1, 101)_ = 0.257, *p* = 0.61; *F*_2(1, 38)_ = 1.465, *p* = 0.23].

In the *Island* comparison, there was a main effect of Condition at region 5 [*F*_1(1, 101)_ = 7.308, *p* < 0.01; *F*_2(1, 38)_ = 6.769, *p* < 0.05] reflecting that participants showed a reading time slowdown in the wh-extraction condition as compared to the no extraction condition. There was no effect of Group at region 5 [*F*_1(1, 101)_ = 0.029, *p* = 0.87; *F*_2(1, 38)_ = 0.173, *p* = 0.68]. There was a marginal interaction in the by-participants analysis, and a significant interaction in the by-items analysis between Group and Condition at region 5 [*F*_1(1, 101)_ = 3.759, *p* = 0.055; *F*_2(1, 38)_ = 9.826, *p* < 0.01]. *Post-hoc t*-tests revealed that native English speakers showed a significant slowdown in the wh-extraction condition as compared to the no extraction condition [*t*_1(53)_ = −3.507, *p* < 0.01, two-tailed paired *t-*test; *t*_2(19)_ = –5.284, *p* < 0.01, two-tailed paired *t-*test]. However, the effect of Condition for Korean learners of English at region 5 was not significant [*t*_1(48)_ = −0.506, *p* = 0.62, two-tailed paired *t-*test; *t*_2(19)_ = 0.317, *p* = 0.75, two-tailed paired *t-*test]. At the spillover region 6, there was no main effect of Condition [*F*_1(1, 101)_ = 0.492, *p* = 0.49; *F*_2(1, 38)_ = 0.067, *p* = 0.80]. There was an effect of Group [*F*_1(1, 101)_ = 13.117, *p* < 0.001; *F*_2(1, 38)_ = 2.696, *p* = 0.11] reflecting slower residual reading times overall for Korean learners of English than for native English speakers. There was no interaction between Group and Condition at region 6 [*F*_1(1, 101)_ = 0.016, *p* = 0.90; *F*_2(1, 38)_ = 0.745, *p* = 0.39]. Thus, subject filled-gap effects emerged only for native English speakers, and only at the critical region in the *Island* comparison.

### Results: Effects of working memory on filled-gap effect size

#### Gap-filling within islands

Regression models for the critical and spillover filled-gap regions (regions 9 and 10) within the relative clause island in *Island* sentences were not significant. For region 9, the first block of the sequential regression was not significant [adjusted *R*^2^ = 0.002, *F*_(3, 99)_ = 1.055, *p* = 0.372]. The addition of the cross-product of Combined Working Memory and Group in the second block did not significantly increase the variance explained by the model [Δ*R*^2^ = 0.000, adjusted *R*^2^ = −0.009, *F*_(1, 98)_ = 0.001, *p* = 0.982]. Similarly, for region 10 the first block of the sequential regression was not significant [adjusted *R*^2^ = 0.008, *F*_(3, 99)_ = 1.267, *p* = 0.29]. The addition of the cross-product of Combined Working Memory and Group in the second block did not significantly increase the variance explained by the model [Δ*R*^2^ = 0.000, adjusted *R*^2^ = −0.002, *F*_(1, 98)_ = 0.000, *p* = 1.00]. Thus, working memory does not predict gap-filling in positions which are subject to island constraints.

#### Gap-filling in grammatically licit positions

As individual differences in working memory may affect the resolution of wh-dependencies in grammatically licensed positions, we also examined whether working memory modulated the magnitude of filled-gap effects in the following positions: the filled object position in *Non-Island* sentences, and the filled subject position in both *Non-Island* and *Island* sentences.

#### Object filled-gap: Non-island sentences

**Figure 5:**
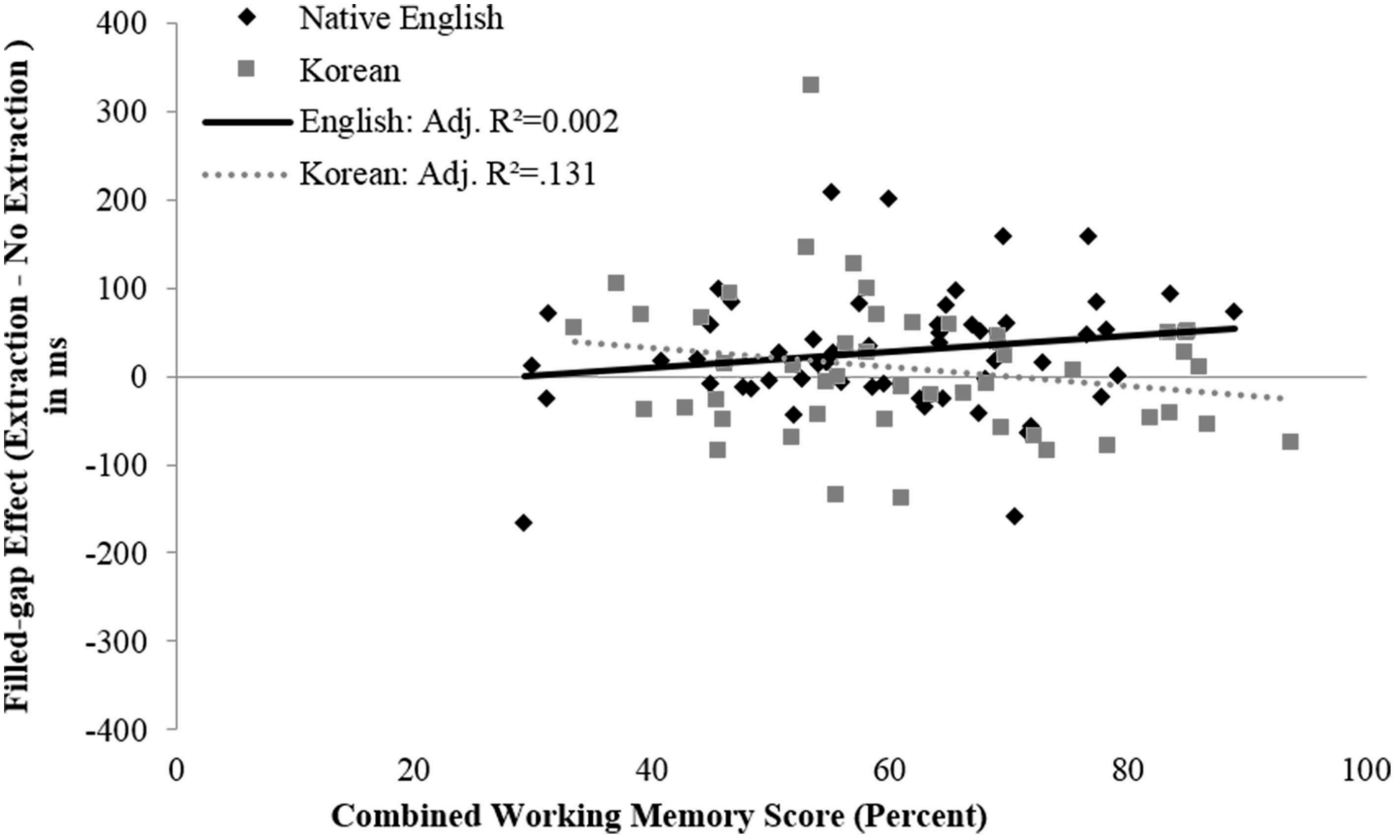
**Filled-gap effect size as a function of combined working memory score for the spillover region (region 9) of the licit object filled-gap position in Experiment 1**. Linear trend lines (least-squares method) are illustrated in the figure; adjusted *R*^2^-values are provided in the legend.

No significant effect of Working Memory on Filled-gap Effect Size was found at the critical region 8 for the object filled-gap. In region 8, the first block of the sequential regression was not significant [adjusted *R*^2^ = 0.016, *F*_(3, 99)_ = 1.539, *p* = 0.209]. The addition of the cross-product of Combined Working Memory and Group in the second block did not significantly increase the variance explained by the model [Δ*R*^2^ = 0.003, adjusted *R*^2^ = 0.008, *F*_(1, 98)_ = 0.285, *p* = 0.595]. A significant effect of Working Memory on Filled-gap Effect Size was found at the spillover region for the object filled-gap. For region 9 the first block of the sequential regression was not significant [adjusted *R*^2^ = −0.001, *F*_(3, 99)_ = 0.949, *p* = 0.42]. However, the addition of the cross-product of Combined Working Memory and Group in the second block significantly increased the variance explained by the model [Δ*R*^2^ = 0.043, adjusted *R*^2^ = 0.034, *F*_(1, 98)_ = 4.584, *p* < 0.05]. Thus, the effect of working memory on object filled-gap effects in the spillover region depends on group membership. In follow-up analyses, the regression slopes were plotted separately by Group (Figure 5). To examine the differences in slope for the two groups, follow-up regression analyses were performed separately for native speakers and learners. The results show that the regression of Working Memory on Filled-gap Effect Size for native speakers, when controlling for age, was not significant [adjusted *R*^2^ = 0.002, *F*_(2, 51)_ = 1.049, *p* = 0.358]. The regression of Working Memory and Age on Filled-gap Effect Size for Koreans was significant [adjusted *R*^2^ = 0.131, *F*_(2, 46)_ = 4.626, *p* < 0.02]. When controlling for age, working memory had a moderately significant effect on Filled-gap Effect Size. For every one standard deviation increase in working memory score, Filled-gap Effect Size decreased by 0.268 standard deviations [*b* = −1.40, *t*_(46)_ = −1.96, *p* = 0.056, β = −0.268, 95% CI (−2.84 −0.036)]. Thus, the data shows a trend suggesting that working memory predicts the degree of Filled-gap Effect Size at the spillover object filled-gap region for Korean learners of English, but not native English speakers. Specifically, an increase in working memory predicts a reduced Filled-gap Effect Size, and thus decreased filled-gap effects, at the spillover region 9 in Korean learners of English.

#### Subject filled-gap: Non-island sentences

For the critical subject filled-gap region in the *Non-Island* sentences (region 5), the first block of the sequential regression was not significant [adjusted *R*^2^ = 0.016, *F*_(3, 99)_ = 1.55, *p* = 0.206]. However, the addition of the cross-product of Combined Working Memory and Group in the second block significantly increased the variance explained by the model [Δ*R*^2^ = 0.084, adjusted *R*^2^ = 0.093, *F*_(1, 98)_ = 9.392, *p* < 0.01]. Thus, the effect of working memory on gap-filling, when controlling for age, depends on Group.

**Figure 6:**
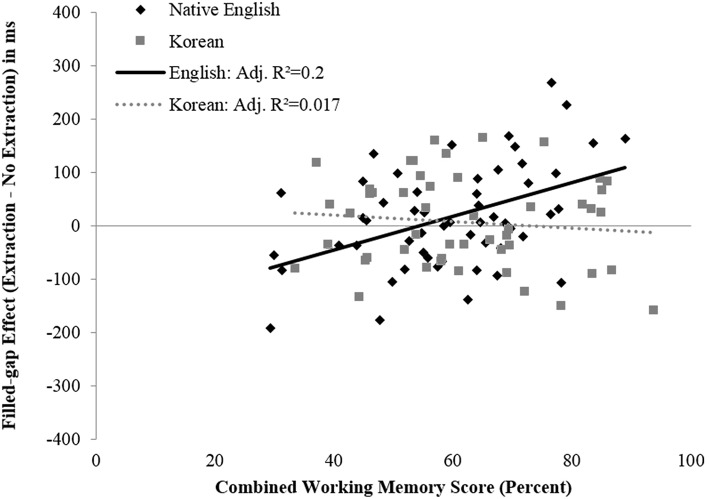
**Filled-gap effect size as a function of combined working memory score for the licit subject filled-gap position (region 5) in Experiment 1**. Linear trend lines (least-squares method) are illustrated in the figure; adjusted *R*^2^-values are provided in the legend.

To better understand the nature of the moderation, the regression slopes were plotted separately by Group (Figure 6). To examine the differences in slope for the two groups at region 5, follow-up regression analyses were performed separately for the two groups. The results show that the regression of Working Memory on Filled-gap Effect Size for native speakers was significant [adjusted *R*^2^ = 0.2, *F*_(2, 51)_ = 7.621, *p* < 0.01]. For every one standard deviation increase in working memory score, Filled-gap Effect Size increased by 0.433 standard deviations, when controlling for age [*b* = 3.029, *t*_(51)_ = 3.506, *p* < 0.01, β = 0.433, 95% CI (1.30–4.76)]. However, the effect of Working Memory on Filled-gap Effect Size for Korean learners of English was not significant [adjusted *R*^2^ = −0.017, *F*_(2, 46)_ = 0.602, *p* = 0.552]. Thus, the data suggests that working memory does predict reading times at the subject filled-gap region for native English speakers, but not for Korean learners of English. Specifically, an increase in working memory predicts an increased slowdown, or filled-gap effect, at the filled subject gap region 5 in native speakers of English.

In the spillover region 6 for the subject filled-gap in the Non-Island sentences, the first block of the sequential regression was not significant [adjusted *R*^2^ = −0.022, *F*_(3, 99)_ = 0.253, *p* = 0.859]. The addition of the cross-product of Combined Working Memory and Group in the second block did not significantly increase the variance explained by the model [Δ*R*^2^ = 0.015, adjusted *R*^2^ = −0.017, *F*_(1, 98)_ = 1.493, *p* = 0.225].

#### Subject filled-gap: Island sentences

For region 5 in the *Island* sentences, the first block of the sequential regression was not significant [adjusted *R*^2^ = 0.012, *F*_(3, 99)_ = 1.425, *p* = 0.24]. The addition of the cross-product of Combined Working Memory and Group in the second block increased the variance explained by the model by a significant amount [Δ*R*^2^ = 0.048, adjusted *R*^2^ = 0.052, *F*_(1, 98)_ = 5.186, *p* < 0.05]. However, follow-up regression analyses performed separately for the two groups found that the regression of Working Memory on Filled-gap Effect Size for Native Speakers, when controlling for age, was not significant [adjusted *R*^2^ = 0.016, *F*_(2, 51)_ = 1.418, *p* = 0.252]. The regression of Working Memory on Filled-gap Effect Size for Korean learners of English, when controlling for age, was also not significant [adjusted *R*^2^ = 0.018, *F*_(2, 46)_ = 1.434, *p* = 0.249]. There were no significant effects in the spillover region 6 in the *Island* sentences. The first block of the sequential regression was not significant [adjusted *R*^2^ = −0.021, *F*_(3, 99)_ = 0.302, *p* = 0.824]. The addition of the cross-product of Combined Working Memory and Group in the second block did not significantly increase the variance explained by the model [Δ*R*^2^ = 0.002, adjusted *R*^2^ = −0.030, *F*_(1, 98)_ = 0.160, *p* = 0.690].

## Discussion

The present study examined whether native speakers and L2 learners show qualitatively similar patterns in the processing of wh-dependencies in both licit and illicit contexts. Previous studies have shown that native speakers attempt to resolve wh-dependencies in grammatically licensed positions but avoid positing gaps in islands (Stowe, 1986; Traxler and Pickering, 1996). In the present study, we replicated this pattern for native English speakers and showed the same pattern of results for advanced Korean learners of English as well. In the non-island sentences, a significant filled-gap effect emerged in the spillover region following the direct object of the verb. A significant interaction with group did not emerge, demonstrating qualitative similarity between the two groups. In Felser et al. (2012), evidence of a filled-gap effect emerged for L2 learners in a later region than the region in which the effect emerged for native speakers, a result which supported their proposal that learners cannot use syntactic information on the same timecourse as native speakers^4^. In contrast, the results of the present study are in line with our previous work, which also showed the same pattern for L2 learners and natives (Aldwayan et al., 2010). While it is true that self-paced reading does not allow the same range of dependent measures as eye-tracking in terms of characterizing the timecourse of processing, it is important to point out that in the Felser et al. (2012) study, the filled-gap effects for natives and learners emerged in distinct regions, not in different dependent measures within the same region.

In contrast to the non-island sentences, where significant object filled-gap effects emerged for both groups, there were no object filled-gap effects in island sentences, in which the critical region was embedded within a relative clause island. Our results are in line with several previous studies which have examined relative clause islands and have shown that learners avoid attempting to resolve wh-dependencies in grammatically unlicensed contexts (Aldwayan et al., 2010; Omaki and Schulz, 2011; Felser et al., 2012; Kim et al., 2015 for Spanish natives)^5^. In the current literature, both studies which showed evidence of gap-filling in islands by L2 learners used a plausibility mismatch paradigm (Boxell and Felser, 2013 in first pass reading measures; Kim et al., 2015 for Korean natives). However, it is important to point out that in the Felser et al. (2012) study, which also used a plausibility mismatch paradigm, learners showed effects of plausibility even earlier than natives but at no point did they show evidence of attempting to resolve wh-dependencies in islands.

Our examination of the subject position yielded a significant subject filled-gap effect for native speakers, but only in island sentences^6^. As we discussed above, this inconsistency across experiments and groups is in line with previous studies. There is an extensive literature discussing why evidence for filled-gap effects in subject position is mixed (Stowe, 1986; Clifton and Frazier, 1989; Clifton and De Vincenzi, 1990; De Vincenzi, 1991; Gibson et al., 1994; Lee, 2004; Johnson, 2015): several researchers have proposed that the adjacency of the wh-filler and the subject position may not provide sufficient time to either generate or commit to a prediction for a subject gap. This proposal would suggest that allowing more time, in terms of the distance between the wh-filler and subject position, may yield different results (see Lee, 2004). Also related to this proposal, one might also expect that individuals with greater processing resources would be more likely to be able to immediately generate a prediction for a subject gap; we will return to this point below in our discussion of individual differences.

The present study also examined the nature of islands by investigating the relationship between working memory and filled-gap effects in both native speakers and L2 learners. A pattern of results showing that individuals with more processing resources are better able to establish wh-dependencies in islands would be compatible with the processing account proposed by Hofmeister and colleagues (e.g., Hofmeister and Sag, 2010). In contrast, grammatical accounts do not predict such a relationship within islands as the parser should simply not predict a gap within island contexts. Note however that a pattern of results that shows no relationship between working memory and filled-gap effects within islands is also potentially compatible with the processing accounts as null results may be explained by a range of factors including, as discussed by Hofmeister et al. (2012a,b, 2013, 2014) inappropriate choice of working memory measures and selection of stimuli that are simply too complex for individual differences to emerge. As our results showed that there was indeed no significant relationship between working memory and filled-gap effects in island contexts for either native speakers or learners, we will consider this range of possibilities as related to our study. In the present study, the lack of a relationship between working memory and filled-gap effects in islands is unlikely to be due to the selection of an inappropriate measure of working memory or lack of statistical power as significant relationships between working memory and filled-gap effects emerged within licit contexts for both learners and natives (although the patterns for the two groups differed). Although the interpretation of these findings is complex, they do suggest, in line with previous studies, that our working memory measure is one that can indeed capture variability in linguistic processing (Daneman and Carpenter, 1980; King and Just, 1991; Just and Carpenter, 1992; Hofmeister et al., 2014; see Hofmeister et al., 2012a for discussion).

Next, we consider whether the difference between the licit and illicit island contexts is simply the result of differences in processing load: if the island sentences simply overwhelmed the parser, perhaps a significant relationship with working memory did not emerge because of a lack of variability. For example, Hofmeister et al. (2014) fail to show a relationship between reading span scores and acceptability judgments for sentences of extreme processing difficulty although significant relationships did emerge for less complex structures. We think that this explanation is unlikely due to the comparability of the stimuli which targeted licit and illicit gap sites (see 9, 10). In both the non-island and island conditions, the target sentences were all grammatical, indirect questions which allowed us to avoid presenting direct wh-questions in isolation, which Hofmeister et al. (2012a) have argued is unnatural. In terms of the comparison between the licit and illicit object positions, it is important to note that these potential gap sites occur at similar points in the sentence (region 8, region 9) and at similar distances from the wh-filler (three and four words after the filler). In addition, in both sentence types, the wh-filler is followed by a single animate noun phrase and a tensed verb. These similarities serve to minimize the differences in processing difficulty of the licit and illicit object gap sites. Thus, while it is difficult to argue categorically in support of or against either account on the basis of a lack of a relationship between working memory and filled-gap effects in islands, we believe the design of the present study can potentially be defended against some of the criticisms raised in the literature by Hofmeister et al. (2012a,b, 2014). In addition, we believe there is merit to the approach we have taken in examining the relationship between individual differences and processing-based dependent measures across both island and non-island contexts. Indeed, it would be interesting to examine whether the results of the current study would be replicated in an experiment testing sentences that include linguistic properties that have been shown to ease the processing of wh-dependencies, such as complex wh-fillers (e.g., Hofmeister and Sag, 2010; Goodall, 2015). Such an experiment would provide an ideal way to address the potential concern that the lack of variability in gap-filling inside islands in the current study could be because the processing of those island structures is simply beyond the reach of all participants, even those with high working memory.

As we discussed earlier, any significant relationships that emerge at the licit gap sites are consistent with both the processing and grammatical accounts of islands but we believe that our findings raise very interesting questions as to the nature of the relationship between working memory and the processing of wh-dependencies in both learners and native speakers. In the non-island sentences, a positive correlation emerged between working memory and the filled-gap effect size at the subject position; this effect was significant only for native speakers. As we discussed above, one possible explanation is that participants with greater processing resources are better able to immediately generate a prediction for a potential gap (e.g., Hutchison, 2007; Slevc and Novick, 2013; Johnson, 2015) and thus show a greater filled-gap effect. The question remains why this relationship did not emerge at the licit subject position in both non-island and island contexts or in the L2 learner group. In a recent study in our lab, Johnson (2015) conducted a large scale study of native speakers (*n* = 110) and intermediate and advanced Korean learners of English (*n* = 100). The self-paced reading experiment included sentences similar to the ones tested in the present study. The results showed that significant subject filled-gap effects emerged for both groups. All participants also completed measures of cognitive abilities including working memory (counting span) and attentional control (number Stroop). The size of the subject filled-gap effect in both natives and L2 learners was significantly related to attentional control, which Hutchison (2007) has argued to be a key component in the ability to generate and maintain predictions. Taken together, these results show that there is even variability in the processing of wh-dependencies that are relatively simple in terms of structure but demanding in terms of the need to automatically generate a prediction for an upcoming gap. This variability may lead to a need for large sample sizes, such as those in Johnson (2015), in order for robust effects to emerge. In addition, in an effort to better understand the cognitive abilities that underlie this variability in both natives and learners, future studies should include a wider range of measures, allowing for a more precise examination of whether the cognitive abilities that underlie variability in native speakers are similar or different to the abilities that underlie the variability in learners.

**Figure 7:**
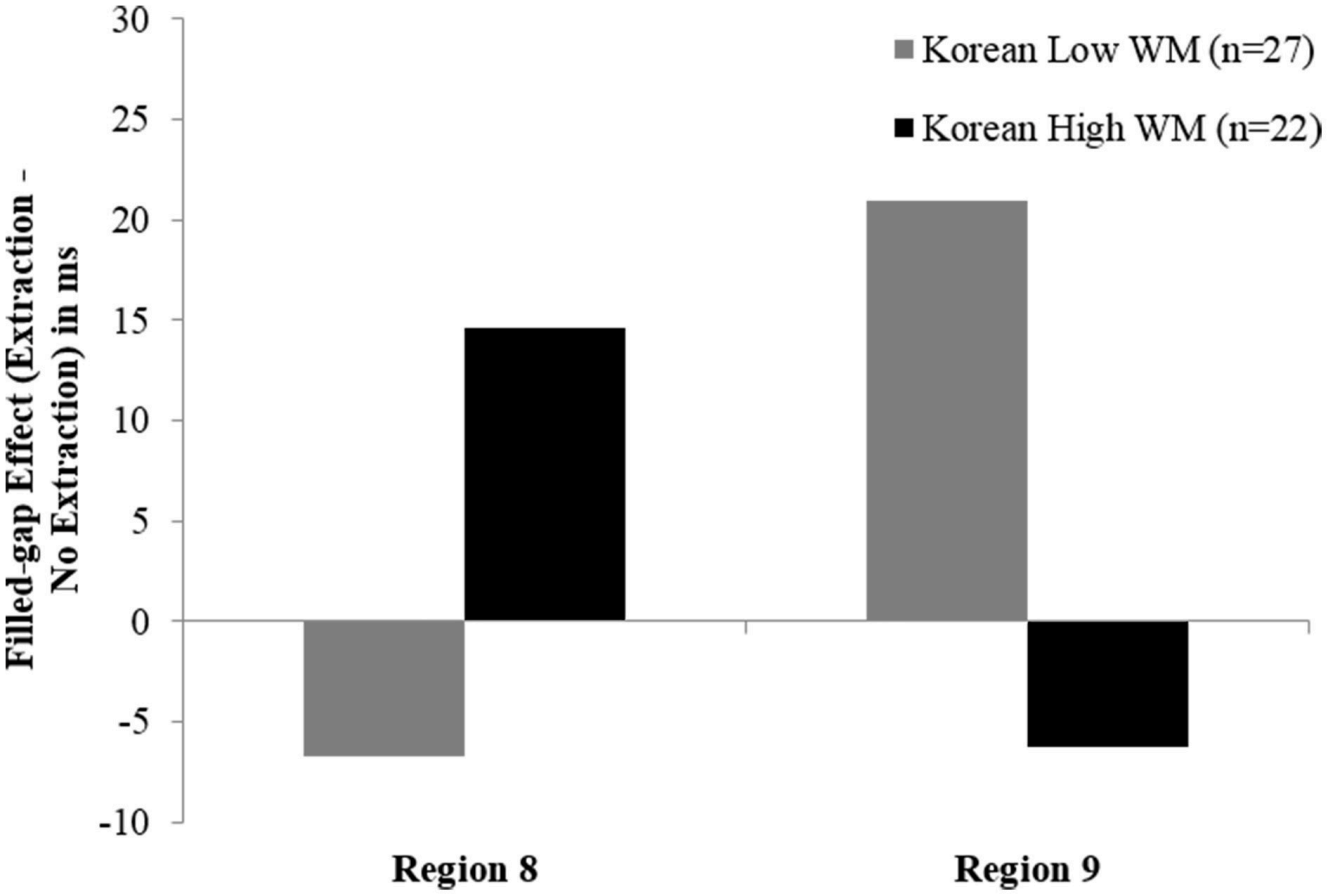
**Filled-gap effect size in high vs. low working memory Korean learners of English at the licit object filled-gap region 8 and its spillover region 9 in Experiment 1**.

Our results for the non-island sentences also showed a relationship between working memory and the size of the licit object filled-gap effect in the spillover region but this effect emerged only for the L2 learners. Unexpectedly, the results showed that an increase in working memory predicted a reduced reading time slowdown or a smaller object filled-gap effect. One possible explanation is that the learners with greater processing resources may have recovered more easily from encountering the filled-gap, resulting in a reduced filled-gap effect at the spillover region. To explore this possibility, we separated the Korean learners of English into high (*n* = 22) and low (*n* = 27) working memory groups, based on whether they scored above or below the mean for the group (62) and then compared the size of the filled-gap effects at both the critical region (region 8) and the spillover region (region 9), where the relationship with working memory emerged (see Figure 7).

This comparison demonstrates that the high working memory group showed a numerical slowdown in the predicted direction only at the critical region. Thus, it is at least possible that learners with higher working memory showed a reduced filled-gap effect at the spillover region because they had already recovered from encountering the lexical material in the preceding region. As this comparison is exploratory, we present this numerical pattern in the learner data in order to suggest a direction for future research, one that may also benefit from an increased sample size, as in Johnson (2015), which may allow a wider range of variability to emerge in both learners and native speakers. An alternative method such as eye-tracking may also allow a more precise characterization of the dynamics of attempting to resolve wh-dependencies, including the initial detection of a filled potential gap site and recovery from this mis-analysis.

Although the results of our individual differences analyses raise many open questions, they suggest that processing resources do modulate the processing of wh-dependencies in certain grammatically licensed contexts. Why different relationships with working memory arise for the learners and native speakers is a very interesting question for future research. Further study is needed to examine whether similar or different cognitive abilities facilitate processing at different points for the two populations.

## Conclusion

In the current study, we investigated the processing of wh-dependencies in both native speakers and L2 learners, examining whether the two groups show qualitatively similar patterns in processing and whether there is a relationship between working memory and filled-gap effects in both island and non-island contexts. The results showed that both native and non-native speakers posit gaps in grammatically licensed contexts but avoid positing gaps in islands. The processing profile of natives and L2 learners was qualitatively similar, showing no evidence of a delay in the use of syntactic knowledge as has been argued in recent proposals (Felser et al., 2012; Boxell and Felser, 2013). Our individual differences analyses showed no relationship between working memory and filled-gap effects within islands but we did observe significant relationships between working memory and the processing of licit wh-dependencies. As the contexts in which these relationships emerged differed for learners and native speakers, our results call for further research examining individual differences in dependency resolution in the two populations.

## Author contributions

All authors listed, have made substantial, direct and intellectual contribution to the work, and approved it for publication.

### Conflict of interest statement

The authors declare that the research was conducted in the absence of any commercial or financial relationships that could be construed as a potential conflict of interest.

## Footnotes

^1^A reviewer suggests that it would have been beneficial to include an offline measure of acceptability. Note that this would primarily be a concern if learners do show filled-gap effects inside islands. However, even if an offline grammaticality judgment task had been included, native-like performance on this type of task would not necessarily indicate a native-like grammar of wh-dependencies (see Aldwayan et al., 2010).

^2^A reviewer asked us to specify the predictions for the relationship between working memory and the size of the filled-gap effect if the Korean learners of English do not in fact show knowledge of syntactic constraints. If the Korean learners of English do not pattern similarly to the native speakers, then the specific nature of the relationship between working memory and the size of the filled-gap effect would need to be examined. For example, if Korean learners of English are found to establish wh-dependencies in islands, and further, if those learners with higher working memory resources showed larger filled-gap effects within islands, then the results would support a processing account. Note, however that, in our results, the Korean learners of English do show similar island sensitivity to the native speakers.

^3^Following a reviewer's suggestion, we examined the relationship between the recall and processing components of the working memory tasks. Recall and accuracy scores for the counting span task were significantly correlated (*r* = 0.300, *p* = 0.002) and those for the reading span task were moderately correlated (*r* = 0.181, *p* = 0.067). While there was a small but positive correlation between these components, we ran new regression models using a new composite working memory score which incorporated participants' performance on both the recall and processing components for each task (using an average of the processing and recall scores), in line with Waters and Caplan (1996). The pattern of results remains unchanged in these new analyses.

^4^A reviewer suggests that the results of the present study may differ from the results of previous studies which showed differences between learners and native speakers because of differences in proficiency levels. The learners in the present study scored between intermediate and advanced levels on the proficiency test and were immersed in the L2 environment. However, the Felser et al. (2012) study also included intermediate-advanced learners who were immersed in an English-speaking environment. As different proficiency measures were used across studies, it is hard to directly compare proficiency levels.

^5^As a reviewer pointed out, the lexical items in the *Non-Island* and the *Island* sentences are not the same, questioning whether this may complicate interpretation of the findings. Our overall finding of a filled-gap effect in the licit object position, and avoidance of a filled-gap effect in object position within an island converges with a range of previous studies using either filled-gap or plausibility manipulations and more closely matched lexical material (e.g., Stowe, 1986; Traxler and Pickering, 1996; Omaki and Schulz, 2011; Felser et al., 2012). However, we agree that a future extension of the current study, with closely matched lexical items across the *Non-Island* and *Island* conditions would be ideal for addressing this open question.

^6^A reviewer pointed out that lexical differences may have played in a role in the distribution of the subject filled-gap effect, which emerged only in the Non-Island comparison. Indeed, as the lexical subject was a proper name in the first comparison but a determiner-noun sequence in the second comparison, this is a possibility. Although very few studies report significant subject filled-gap effects, across studies, significant effects have been reported for both determiner-noun sequences (e.g., Aldwayan et al., 2010) and proper names (e.g., Lee, 2004; Johnson, 2015). Moreover, not all studies which include a proper name in subject position show significant subject filled-gap effects (e.g., Stowe, 1986). Thus, the effect of the structure of the noun phrase remains an interesting open question.
