# Supplementary material for: Syntactic Constraints and Individual Differences in Native and Non-Native Processing of Wh-Movement
Source: Front Psychol. 2016 Apr 22;7:549. doi: 10.3389/fpsyg.2016.00549 (PMC4840386; doi:10.3389/fpsyg.2016.00549)
Supplement: Supplementary file 1 [file DataSheet1.DOCX]

Stimuli: Non-Island Sentences

1a. My brother asked if Barbara will photograph Ali beside Mom at the graduation.

1b. My brother asked who Barbara will photograph Ali beside at the graduation.

2a. My niece guessed if Kelly will photograph Kim with Edward at the parade.

2b. My niece guessed who Kelly will photograph Kim with at the parade.

3a. My sister knew if Roger will place Pat with Jason at the lunchtable.

3b. My sister knew who Roger will place Pat with at the lunchtable.

4a. My nephew revealed if Alex will put Ted near Nancy at the gathering.

4b. My nephew revealed who Alex will put Ted near at the gathering.

5a. My friend wondered if Julie will recommend Amy to Sarah before the deadline.

5b. My friend wondered who Julie will recommend Amy to before the deadline.

6a. My mother asked if John will find Rob beside Dad at the restaurant.

6b. My mother asked who John will find Rob beside at the restaurant.

7a. My aunt guessed if Patrick will film Sue with Kelly at the banquet.

7b. My aunt guessed who Patrick will film Sue with at the banquet.

8a. My grandmother knew if Adam will find Jen with Rachel at the mall.

8b. My grandmother knew who Adam will find Jen with at the mall.

9a. My classmate revealed if Jack will meet Moe with Sarah before the dance.

9b. My classmate revealed who Jack will meet Moe with before the dance.

10a. My cousin wondered if David will put Liz near Jack at the wedding.

10b. My cousin wondered who David will put Liz near at the wedding.

11a. The manager asked if Ethan will meet Sam with Jeff outside the office.

11b. The manager asked who Ethan will meet Sam with outside the office.

12a. The student guessed if Ryan will introduce Jim to Heather after the break.

12b. The student guessed who Ryan will introduce Jim to after the break.

13a. The teachers knew if Michael will discover Ron with Jerry during the game.

13b. The teachers knew who Michael will discover Ron with during the game.

14a. The secretary revealed if Shawn will introduce Lou to Jared after the speech.

14b. The secretary revealed who Shawn will introduce Lou to after the speech.

15a. The instructor wondered if Chris will film Tom with Susan at the reception.

15b. The instructor wondered who Chris will film Tom with at the reception.

16a. The boy asked if Matt will place Ben with Susie at the party.

16b. The boy asked who Matt will place Ben with at the party.

17a. The babysitter guessed if Christopher will discover Dan with Lindsey in the closet.

17b. The babysitter guessed who Christopher will discover Dan with in the closet.

18a. The manager knew if Katie will recommend Joe to Patricia after the assembly.

18b. The manager knew who Katie will recommend Joe to after the assembly.

19a. The girl revealed if Melissa will seat Ann by Susan at the dinner.

19b. The girl revealed who Melissa will seat Ann by at the dinner.

20a. The teacher wondered if Harry will seat Bob by Rachel in the classroom.

20b. The teacher wondered who Harry will seat Bobby in the classroom.

Stimuli: Island Sentences

1a. My father asked if the actress that married Tyler last summer kissed the director during the rehearsal.

1b. My father asked who the actress that married Tyler last summer kissed during the rehearsal.

2a. My manager investigated if the assistant that fired Kylie last June seduced the supervisor before the party.

2b. My manager investigated who the assistant that fired Kylie last June seduced before the party.

3a. My brother questioned if the journalist that followed Henry last Saturday provoked the guard at the store.

3b. My brother questioned who the journalist that followed Henry last Saturday provoked at the store.

4a. My teacher wondered if the principal that suspended Jacob last spring disappointed the parents with the news.

4b. My teacher wondered who the principal that suspended Jacob last spring disappointed with the news.

5a. My brother asked if the woman that defended Dylan last Tuesday slapped the thief on the face.

5b. My brother asked who the woman that defended Dylan last Tuesday slapped on the face.

6a. The psychologist investigated if the boy that hit Timmy last Thursday offended the teacher after the incident.

6b. The psychologist investigated who the boy that hit Timmy last Thursday offended after the incident.

7a. My uncle questioned if the man that visited Ellie last night irritated the neighbors with the noise.

7b. My uncle questioned who the man that visited Ellie last night irritated with the noise.

8a. My wife wondered if the hunter that located Jenny last Sunday contacted the police from the camp.

8b. My wife wondered who the hunter that located Jenny last Sunday contacted from the camp.

9a. My daughter asked if the clown that scared Eddie last Wednesday delighted the nanny with the balloon.

9b. My daughter asked who the clown that scared Eddie last Wednesday delighted with the balloon.

10a. The prosecutor investigated if the accountant that fooled Maria last December defrauded the investors over the internet.

10b. The prosecutor investigated who the accountant that fooled Maria last December defrauded over the internet.

11a. The senator questioned if the traitor that exposed Diana last month betrayed the president after the scandal.

11b. The senator questioned who the traitor that exposed Diana last month betrayed after the scandal.

12a. My nephew wondered if the banker that dated Molly last year shocked the auditor with the report.

12b. My nephew wondered who the banker that dated Molly last year shocked with the report.

13a. The politician asked if the reporter that challenged Carol last Monday annoyed the moderator at the debate.

13b. The politician asked who the reporter that challenged Carol last Monday annoyed at the debate.

14a. The sheriff investigated if the boxer that defeated Peter last March paid the referee for the championship.

14b. The sheriff investigated who the boxer that defeated Peter last March paid for the championship.

15a. The reporter questioned if the politician that impressed Peggy last February insulted the senator at the conference.

15b. The reporter questioned who the politician that impressed Peggy last February insulted at the conference.

16a. The agent wondered if the producer that consulted Lucas last Friday hired the musician after the audition.

16b. The agent wondered who the producer that consulted Lucas last Friday hired after the audition.

17a. The chief asked if the officer that interviewed James last week angered the lawyer during the trial.

17b. The chief asked who the officer that interviewed James last week angered during the trial.

18a. The doctor investigated if the nurse that vaccinated Aaron last April harmed the child at the hospital.

18b. The doctor investigated who the nurse that vaccinated Aaron last April harmed at the hospital.

19a. The director questioned if the singer that bothered Becky last season criticized the pianist after the concert.

19b. The director questioned who the singer that bothered Becky last season criticized after the concert.

20a. The agent wondered if the spy that shot Megan last evening kidnapped the ambassador from the hotel.

20b. The agent wondered who the spy that shot Megan last evening kidnapped from the hotel.
